# The burden and social determinants of asthma for adults in the state of Georgia

**DOI:** 10.21633/jgpha.6.406

**Published:** 2017

**Authors:** Mark Ebell, Christian Marchello, Jean O'Connor

**Affiliations:** 1 Department of Epidemiology, College of Public Health, University of Georgia, Athens, GA; 2 Chronic Disease Prevention Director, Georgia Department of Public Health, Atlanta, GA

**Keywords:** Asthma, prevalence, social determinants of health, access to care

## Abstract

**Background:**

Asthma is a serious chronic health condition, and social determinants may affect its prevalence.

**Methods:**

Data from the Behavioral Risk Factors Surveillance Survey (BRFSS), the Georgia Asthma Call-back Survey (ACBS), and the Georgia hospital and emergency department survey for patients with a diagnosis of asthma were used. All data were from the years 2011 through 2014. SAS and SUDAAN software were used to calculate weighted prevalence estimates and to perform univariate and multivariate analyses of the association between social determinants, other risk factors, and asthma outcomes.

**Results:**

The prevalence of asthma was highest among non-Hispanic blacks, women, and persons with less than a high school education, with an annual household income below $25,000, and in rural parts of the state (south and northwest Georgia). Those without insurance for more than three years had a higher prevalence of asthma than those who had insurance or had been uninsured less than 6 months. Although the percentage without insurance declined from 2012 to 2014, more than 1 in 5 adults of working age with asthma still lacked health insurance, and more than half had been without it for more than 3 years. One-third of Georgians with asthma could not see a doctor, at least on one occasion, because of cost, and more than a third were currently paying off medical bills. Approximately one quarter did not report having a personal physician, and a similar percentage reported having more than one year since their last check-up. In multivariate analyses, women (adjusted odds ratio [aOR] 1.61), smokers (aOR 1.54), and persons with a higher BMI (aOR 1.56) were all independently associated with having asthma.

**Conclusions:**

For the state of Georgia, there are associations between social determinants, such as education, income, and geography, and the prevalence of asthma, and many patients lack access to care. Addressing social determinants, including having affordable health insurance, is necessary to improve management of asthma.

## Introduction

Asthma, a chronic respiratory disease characterized by inflammation and bronchospasms, affects both children and adults. Typical symptoms include wheezing, dyspnea, and tachypnea. Characteristics of the disease are frequent exacerbations, which may require systemic therapy and hospitalization. Based on data from the National Health Interview Survey conducted by the Centers for Disease Control and Prevention (CDC), the prevalence of asthma in the United States is 6.3% among males and 9.0% among females (“CDC Asthma Surveillance: Data, statistics and surveillance,” 2013). In addition, about half of children with asthma in the United States miss at least one school day each year, accounting for 13.8 million missed school days in 2013 (“Asthma Stats: Asthma-related missing school days among children 5 to 17 years,” 2013).

Various social determinants of health affect the burden of asthma, which falls disproportionately on women and African-Americans. These include tobacco use in the home, neighborhood disorders, environmental pollution, pests in the home, stress, socioeconomic status, parental mental health problems, country of origin, and family structure (Cruz, Bateman, & Bousquet, 2010; Sembajwe et al., 2010; Shiue, 2013; Victorino & Gauthier, 2009; Vo, Bair-Merritt, Camargo, Eisenberg, & Long, 2016; Williams, Sternthal, & Wright, 2009). These factors affect the prevalence of asthma, its clinical outcomes, and the likelihood of complications.

In the current study, the most recent surveillance data were used to report the burden of asthma in the state of Georgia. Also reported are the effects of social determinants of health, including socioeconomic status, race, rurality, usual source of healthcare, insurance status, and education, on the prevalence of asthma.

## Methods

Since this was a secondary analysis of de-identified, publicly available data under a contract with the Georgia Department of Public Health, institutional review board approval was not required. Data sources for the state of Georgia were from the Behavioral Risk Factors Surveillance Survey (BRFSS), the Georgia Asthma Call-back Survey (ACBS), and the Georgia hospital and emergency department survey for patients with a diagnosis of asthma. All data were from the years 2011 to 2014.

The BRFSS is an annual, stratified, random-digit dial telephone interview conducted by the CDC (http://www.cdc.gov/brfss/). Participants are non-institutionalized Georgia residents aged 18 years or older who are asked about their health-related behaviors, chronic conditions, and medical coverage. With the BRFSS, weighted prevalence estimates of asthma among Georgia residents were calculated by use of SAS 9.4 and SUDAAN 11.0.1 software. In addition to common determinants of interest (e.g., sex, age, education), the association of asthma with certain healthcare related variables were also calculated. Univariate and multivariate logistic regressions were performed with SUDAAN to determine the adjusted odds ratios (aOR) of selected variables for the risk of having asthma.

The ACBS is a survey conducted two weeks after a participant has responded to the BRFSS (http://www.cdc.gov/brfss/acbs/). In the BRFSS, if the participant answered ‘yes’ to ever having been diagnosed with asthma, they become eligible for the ACBS but must have given consent for the follow-up call. The ACBS is used to gather additional information from asthma participants regarding their control measures, effect on daily living, occupation, and environment. Weighted prevalence estimates of asthma were calculated using SAS and SUDAAN software.

De-identified data regarding hospital inpatient discharge and emergency department (ED) visits, collected by the Georgia Department of Public Health from non-federal acute care hospitals in Georgia were used to determine the burden of patients hospitalized or seen in EDs with a primary diagnosis of asthma (ICD-9 493.0 - 493.9).

## Results

**Figure 1:**
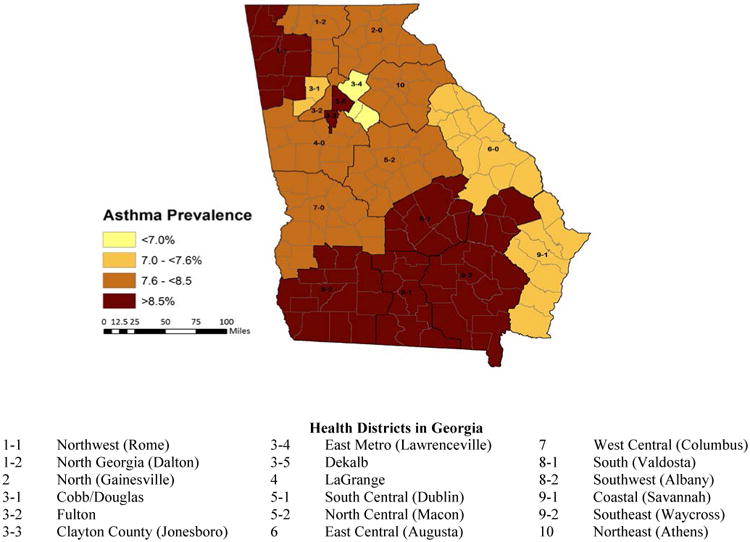
Asthma prevalence by public health district in the state of Georgia

Table 1 summarizes the prevalence of asthma by sex, age, race, and other demographic factors by year. The prevalence of asthma was highest among non-Hispanic blacks, and lowest among Hispanics, although there was a secular trend of increasing prevalence among Hispanics between 2011 and 2014. In 2014, asthma was more prevalent for women (10.0%; 95% CI: 8.7%-11.4%) than for men (6.7%; 95% CI: 5.5%-8.2%) and among persons with less than a high school education (12.0%; 95% CI: 9.2%-15.6%) than college graduates (6.7%; 95% CI: 5.5%-8.1%). An annual household income below $25,000 was also associated with a higher prevalence of asthma. Figure 1 shows the prevalence of asthma by health districts from 2012 to 2014 (a list of districts is also shown). The prevalence was higher in south and northwest Georgia than in other parts of the state.

Regarding clinical and lifestyle factors (Table 2), the prevalence of asthma in 2014 was higher among respondents who were current smokers, who had a body mass index (BMI) > 30 kg/m^2^, and who did not exercise outside of work. It was also higher among those who had poor self-reported physical health (13.4%; 95% CI: 11.6%-15.4% vs 5.7%; 95% CI: 4.8%-6.9%), those with poor self-reported mental health (11.7%; 95% CI: 10.0%-13.8% vs 6.5%; 95% CI: 5.6%-7.7%), and those whose activities were limited due to physical, mental, or emotional problems (17.1%; 95% CI: 14.6%-19.9% vs 6.4%; 95% CI: 5.5%-7.5%).

Table 3 summarizes data regarding the prevalence of asthma by healthcare access, coverage, and satisfaction with care. Those who had been without coverage for more than three years in 2014 had a higher prevalence of asthma (12.5%; 95% CI: 8.3%-18.3%) than patients who were insured or had been uninsured for less than 6 months. Among persons with asthma (Table 4), the percentage of those aged 18 to 64 years without insurance declined from 2012 to 2014, but more than 1 in 5 adults in Georgia with asthma still lacked health insurance. In addition, more than half (54.6%) of those with asthma who did not have insurance had been without it for more 3 years. Each year, about one-third of Georgians with asthma were unable to see a doctor at least once because of cost; a similar percentage each year delayed getting care for reasons other than cost, and more than a third were currently paying off medical bills. Approximately one quarter did not report having a personal physician, and asimilar percentage reported having more than one year since their last check-up. Nevertheless, most asthma patients were either very satisfied (54.7%) or somewhat satisfied (38.9%) with the healthcare that they received. Utilization was high, with 40.8% reporting more than five doctor visits in the previous year.

Table 5 summarizes the univariate logistical regression analyses. Women had a 1.53 (95% CI: 1.18-1.99) times higher odds of asthma than men, but race and age did not show any significant differences between groups. Higher income and education levels decreased odds of asthma (OR 0.60 if >$75k income; OR 0.52 if college graduate). Regarding lifestyle, individuals who reported not exercising in the last 30 days, who were smokers, or who had physical or mental problems had increased odds of asthma.

In the multivariate logistical regression analyses that controlled for socioeconomic variables, smoking, and BMI, education and race were not significantly associated with prevalence of asthma (Table 6). Women (adjusted odds ratio [aOR] 1.61), smokers (aOR 1.54), and persons with a higher BMI (aOR 1.56) were independently associated with having asthma. Although higher income was generally associated with lower odds of asthma, those with incomes greater than $75,000 per year were not significantly different from those with incomes less than $15,000 per year (OR 0.77; 95% CI: 0.46-1.29). However, those with incomes ranging from $25,000 to $74,000 per year had decreased adjusted odds of asthma relative to those with incomes less than $15,000 per year (0.49 to 0.58 versus 1.0, the referent).

In Georgia during 2014, there were 6,601 asthma-related hospitalizations of adults, a rate of 87 per 100,000 and a decrease from 2012 of about 12 per 100,000. ED visits also decreased slightly from 443 per 100,000 (31,929 total ED visits) to 415 per 100,000 (31,595 total ED visits). Although both the total number and rate of ED visits decreased from 2012, the charges for these visits increased by $14.2 million between 2012 and 2014, from $68.1 million to $82.3 million. The rate for hospitalization of women (121/100,000) due to asthma was more than two times the rate for men (49/100,000). The rate of hospitalizations increased as age increased (25/100,000 for those 18-24 years vs 149/100,000 for those 65+ years), whereas ED visits decreased with increasing age (547/100,000 for those 18-24 years vs 195/100,000 for those 65+ years). The rates of hospitalizations and ED visits for African Americans were two and four times higher than rates for Whites, respectively.

## Discussion

Asthma creates a serious health burden for the state of Georgia, and the impact varies by age, sex, race, educational attainment, and insurance status. The prevalence of asthma decreased with age, although rates of hospitalization and ED use increased, and asthma was more common for patients uninsured for more than 3 years and those with less educational attainment. Asthma was also more common for persons who smoke, those who are obese, and those who did not exercise.

Some of the univariate associations may represent reverse causality (such as the association with exercise), confounding, or effect modification. Multivariate analyses found that female sex, being a current smoker, and having a BMI >= 30 kg/m2 were independent predictors of having asthma. After adjustment for other clinical variables, race and educational attainment were no longer predictors of asthma, and incomes between $25,000 and $74,000 per year were protective. This may be because smoking is more common in persons with lower levels of educational attainment, and lower educational attainment is associated with lower income (Gilman et al., 2008).

Regarding secular trends from 2011 to 2014, the overall prevalence of asthma remained relatively constant. The prevalence of asthma among Hispanics increased from 3.5% to 9.0% (the latter value similar to that of other groups). This may be due to greater health care access following implementation of the Affordable Care Act and leading to greater ascertainment of disease that had previously been present in the population but had been undiagnosed. Asthma was more prevalent in south and northwest Georgia, rural parts of the state that suffer from challenges to healthcare access and the availability of primary care physicians.

There were some favorable trends. For example, there was a trend toward fewer patients being uninsured, likely due to passage of the Affordable Care Act in 2009 and its rollout across the country in subsequent years. However, many patients with asthma were still uninsured, a problem that could be addressed by expanding access to Medicaid in Georgia. There were also fewer hospitalizations and ED visits, but overall costs increased. The price of metered dose inhalers, even for drugs long on the market, has increased, with out-of-pocket costs rising from $13.60 to $25.00 after the ban on chlorofluorocarbon led to reformulation and the loss of generic inhalers (Jena, Ho, Goldman, & Karaca-Mandic, 2015).

The results have implications for public health and medical systems in the state of Georgia. Approximately a third of respondents with asthma had delayed seeking care, had not seen a doctor in the past 12 months, or were paying off medical bills. These factors create barriers to appropriate primary care and specialty management of asthma that could prevent hospitalizations and ED visits. Potential solutions include broader access to health insurance, including Medicaid; provision of self-management education; and support for more federally qualified health centers and primary care residency training programs that accept patients with Medicaid as well as those without insurance. These efforts are particularly relevant in the rural south and northwest parts of the state, where there is a shortage of primary care physicians.

## Conclusions

In Georgia, asthma is independently associated with female sex, obesity, and being a current smoker. Higher rates of tobacco use may mediate the associations with lower educational attainment and low income. The prevalence is also higher in south and northwest Georgia, which are generally rural parts of the state.

## Figures and Tables

**Table 1:** Prevalence of asthma by key demographic variables based on BRFSS data

Variable	2011	2012	2013	2014	2011-2014
**Number of participants**	9895	6068	8082	6309	30,354
Overall asthma prevalence (95% CI)*	9.6%	8.2%	8.4%	8.4%	8.7%
**Sex**					
Male^a^	7.5%	5.1%	5.2%	6.7%	6.1%
Female	11.6%¥	11.0%¥	11.3%¥	10.0%¥	11.0%¥
**Race**					
White, non-Hispanic^a^	9.9%	7.7%	7.9%	8.5%	8.5%
Black, non-Hispanic	10.9%	10.7%	10.5%	8.0%	10.0%¥
Hispanic	3.5%¥	3.4%¥	5.9%	9.0%	5.5%¥
Other, non-Hispanic	7.8%	6.2%	6.6%	9.8%	7.6%
**Age**					
18-24^a^	10.0%	9.3%	13.0%	9.3%	10.4%
25-34	10.6%	7.8%	6.7%¥	7.2%	8.1%
35-44	9.3%	6.6%	7.9%¥	8.1%	8.0%
45-54	9.3%	9.0%	7.3%¥	8.0%	8.4%
55-64	9.1%	9.0%	9.0%	9.9%	9.3%
65+	9.3%	7.8%	7.7%¥	8.3%	8.3%
**Income (yearly)**					
<$15k^a^	16.0%	14.4%	18.0%	12.5%	15.2%
$15k-$24k	12.0%	10.9%	8.9%¥	11.6%	10.9%¥
$25k-$34k	8.6%¥	7.5%¥	8.8%¥	5.9%¥	7.8%¥
$35k-$49k	8.8%¥	4.6%¥	6.0%¥	6.6%¥	6.5%¥
$50k-$74k	7.7%¥	5.9%¥	5.7%¥	5.8%¥	6.3%¥
>$75k	6.5%¥	5.6%¥	5.4%¥	7.8%	6.4%¥
**Education**					
Did not graduate HS^a^	13.4%	12.2%	11.0%	12.0%	12.2%
Graduated HS	9.8%	7.4%¥	8.2%	8.1%	8.4%¥
Some college	9.0%	8.9%	8.3%	8.2%	8.6%¥
Graduated college	7.4%¥	5.8%¥	7.0%¥	6.7%¥	6.7%¥

* All prevalence estimates are weighted.

a Reference for group.

¥ Prevalence estimate is significantly different from reference, based on non-overlapping 95% confidence intervals.

**Table 2:** Asthma prevalence by clinical and lifestyle factors

Variable	2011	2012	2013	2014	2011-2014
**Current smoker**					
Yes^a^	13.5%	9.8%	10.4%	13.1%	11.7%
No	8.5%¥	7.6%	8.1%	7.8%¥	8.0%¥
**Body mass index (kg/m^2^)**					
<25^a^	8.4%	6.6%	7.6%	7.0%	7.4%
>=25 and <30	8.3%	7.3%	7.5%	7.4%	7.6%
>=30	12.9%¥	10.5%¥	10.8%¥	10.7%¥	11.2%¥
**Exercise or other physical activity in last 30 days other than job**					
Yes^a^	8.6%	7.5%	7.6%	7.6%	7.8%
No	12.2%¥	10.5%¥	10.3%¥	11.0%¥	11.0%¥
**Activities limited because of physical, mental, or emotional problems**					
Yes^a^	20.3%	15.9%	16.1%	17.1%	17.5%
No	6.2%¥	6.1%¥	6.6%¥	6.4%¥	6.3%¥
**Reported physical health was not good in the last 30 days**					
Yes^a^	17.7%	14.9%	13.5%	13.4%	14.8%
No	5.7%¥	4.9%¥	5.6%¥	5.7%¥	5.5%¥
**Reported mental health was not good in the last 30 days**					
Yes^a^	15.5%	12.6%	12.6%	11.7%	13.1%
No	6.5%¥	6.1%¥	6.4%¥	6.5%¥	6.4%¥

a Reference for group

¥ Prevalence estimate is significantly different from reference, based on non-overlapping 95% confidence intervals.

**Table 3:** Asthma prevalence by healthcare access, coverage, and satisfaction. Data not available for 2011 and 2012 for some variables

Variable	2011	2012	2013	2014	2011-2014 or 2013-2014 * (95% CI)
**Primary source of health care coverage**					
Employer or union			6.9%	6.9%	6.9% (6.1%-7.9%)
Buys own			6.1%	6.8%	6.5% (4.7%-8.8%)
Medicare			n/a	12.4%	12.4% (10.3%-14.9%)
Medicaid			17.8%	12.9%	16.2% (12.5%-20.6%)
Tricare			8.0%	7.5%	7.8% (5.5%-11.1%)
Other source			7.9%	13.8%	9.9% (6.9%-13.9%)
None			9.6%	19.6%	10.1% (7.5%-13.4%)
**Consider one person as personal doctor or health care provider**					
Yes, only 1	9.8%	8.2%	8.5%	8.6%	8.8% (8.3%-9.3%)
More than 1	13.1%	11.7%	9.2%	11.3%	11.3% (9.7%-13.0%)
No	8.2%	7.0%	7.7%	7.3%	7.6% (6.6%-8.6%)
**Length of time since last check up**					
Within the last year	10.2%	8.4%	8.4%	8.5%	8.9% (8.4%-9.4%)
More than 1 year, less than 2 years	8.1%	7.4%	8.7%	9.0%	8.3% (7.0%-9.8%)
More than 2 years, less than 5 years	7.2%	9.0%	8.7%	8.7%	8.4% (6.8%-10.4%)
5 or more years ago	10.0%	7.5%	7.3%	6.6%	7.8% (6.1%-9.9%)
**Did not have health coverage in last 12 months**					
Yes			10.9%	6.5%	8.4% (6.2%-11.2%)
No			7.9%	8.8%	8.3% (7.6%-9.1%)
**How long since last had health coverage**					
6 months or less			5.5%	6.0%	5.7% (3.3%-9.7%)
More than 6 months, less than 1 year			11.8%	0.4%	6.7% (3.5%-12.5%)
More than 1 year, less than 3 years			7.2%	7.9%	7.6% (5.0%-11.4%)
More than 3 years			13.4%	12.5%	13.0% (10.1%-16.5%)
Never			4.2%	7.6%	5.9% (3.4%-10.1%)
**Number of doctor visits in last 12 months**					
1			5.7%	4.5%	5.1% (3.9%-6.7%)
2			7.2%	4.8%	6.0% (4.8%-7.4%)
3			5.9%	9.6%	7.8% (6.2%-9.7%)
4			8.4%	8.7%	8.6% (6.9%-10.6%)
5			11.5%	8.4%	10.0% (7.6%-13.1%)
>5			14.5%	15.9%	15.2% (13.5%-17.1%)
**Satisfaction with health care received**					
Very satisfied			7.6%	7.6%	7.6% (6.9%-8.4%)
Somewhat satisfied			9.7%	9.9%	9.8% (8.6%-11.1%)
Not at all satisfied			12.3%	11.9%	12.1% (9.1%-10.6%)

* Results shown are for 2013-2014 where data were not available for 2011 and 2012.

n/a – Medicare was not an option for respondents in 2013.

**Table 4:** Healthcare access, coverage, and satisfaction for patients with asthma

Variable	2011	2012	2013	2014	2013-2014
**Any form of insurance respondents aged 18-64**					
Yes	71.7%	65.8%	70.4%	77.7%	73.9%
No	28.3%	34.2%	29.6%	22.3%	26.1%
**Primary source of health care coverage**					
Employer or union	n/a	n/a	48.5%	43.5%	46.0%
Buys on own	n/a	n/a	9.3%	9.4%	9.4%
Medicare	n/a	n/a	20.2%	31.4%	25.8%
Medicaid	n/a	n/a	n/a	6.7%	6.7%
Tricare	n/a	n/a	5.9%	3.6%	4.8%
Other source	n/a	n/a	5.8%	4.6%	5.2%
None	n/a	n/a	10.3%	0.8%	5.6%
**Could not see doctor because of cost**					
Yes	33.9%	33.1%	32.5%	27.8%	30.1%
No	66.1%	66.9%	67.5%	72.2%	69.9%
**Delayed getting medical care reasons other than cost in the last 12 months**					
Yes	n/a	n/a	38.0%	26.9%	32.4%
No	n/a	n/a	62.0%	73.1%	67.6%
**Consider one person as personal doctor or health care provider**					
Yes, only 1	66.6%	66.1%	64.3%	66.9%	65.6%
More than 1	10.5%	12.6%	9.7%	8.3%	9.0%
No	22.9%	21.4%	26.0%	24.8%	25.4%
**Length of time since last check up**					
Within the last year	76.4%	72.6%	73.4%	73.6%	73.5%
More than 1 year, less than 2 years	10.0%	12.1%	11.5%	12.4%	12.0%
More than 2 years, less than 5 years	6.2%	9.1%	7.8%	8.3%	8.0%
5 or more years ago	7.4%	6.3%	7.4%	5.6%	6.5%
**Did not have health coverage in last 12 months**					
Yes	n/a	n/a	9.1%	7.0%	8.0%
No	n/a	n/a	90.9%	93.0%	92.0%
**How long since last had health coverage**					
6 months or less	n/a	n/a	7.9%	9.0%	8.4%
More than 6 months, less than 1 year	n/a	n/a	12.4%	0.4%	7.0%
More than 1 year, less than 3 years	n/a	n/a	14.3%	18.0%	16.0%
More than 3 years	n/a	n/a	56.3%	52.4%	54.6%
Never	n/a	n/a	9.1%	20.2%	14.1%
**Number of doctor visits in last 12 months**					
1	n/a	n/a	14.0%	10.4%	12.2%
2	n/a	n/a	17.4%	11.7%	14.5%
3	n/a	n/a	10.8%	17.9%	14.3%
4	n/a	n/a	11.3%	11.6%	11.5%
5	n/a	n/a	8.1%	5.3%	6.7%
>5	n/a	n/a	38.4%	43.1%	40.8%
**Satisfaction with health care received**					
Very satisfied	n/a	n/a	54.7%	54.8%	54.7%
Somewhat satisfied	n/a	n/a	38.9%	38.0%	38.4%
Not at all satisfied	n/a	n/a	6.5%	7.2%	6.9%
**Currently paying off medical bills**					
Yes	n/a	n/a	31.3%	34.1%	32.7%
No	n/a	n/a	68.7%	65.9%	67.3%

**Table 5:** Univariate Analyses of Selected Variables on Odds for Having Asthma

Variable	OR	Lower 95% CI	Upper 95% CI	p-value
**Sex**				
Male	1.00			reference
Female	1.53	1.18	1.99	<0.01
**Race**				
White, non-Hispanic	1.00			reference
Black, non-Hispanic	0.94	0.71	1.24	0.67
Hispanic	1.06	0.62	1.84	0.82
Other, non-Hispanic	1.17	0.60	2.28	0.65
**Age**				
18-24	1.00			reference
25-34	0.76	0.44	1.32	0.34
35-44	0.86	0.51	1.46	0.58
45-54	0.85	0.52	1.40	0.53
55-64	1.08	0.68	1.71	0.76
65+	0.89	0.57	1.40	0.61
**Income**				
<$15k	1.00			reference
$15k-$24k	0.92	0.62	1.36	0.68
$25k-$34k	0.44	0.26	0.74	<0.01
$35k-$49k	0.50	0.03	0.81	0.01
$50k-$74k	0.43	0.27	0.70	<0.01
>$75k	0.60	0.40	0.89	0.01
**Education**				
Did not graduate HS	1.00			reference
Graduated HS	0.65	0.44	0.94	0.02
Some college	0.65	0.44	0.96	0.03
Graduated college	0.52	0.36	0.75	<0.01
**Current smoker**				
No	1.00			reference
Yes	1.77	1.30	2.41	<0.01
**Body mass index (kg/m2)**				
<25	1.00			reference
>=25 and <30	1.06	0.76	1.48	0.73
>=30	1.58	1.15	2.16	<0.01
**Exercise or other physical activity in last 30 days other than job**				
Yes	1.00			reference
No	1.51	1.16	1.96	<0.01
**Limited activities because of physical, mental, or emotional problems**				
No	1.00			reference
Yes	3.01	2.33	3.88	<0.01
**Reported physical health was not good in the last 30 days**				
No	1.00			reference
Yes	2.54	1.97	3.29	<0.01
**Reported mental health was not good in the last 30 days**				
No	1.00			reference
Yes	1.90	1.48	2.44	<0.01

OR = odds ratio; CI = confidence interval

**Table 6:** Multivariate Analysis of Socioeconomic Variables, Smoking, and BMI on Odds of Asthma

Variable	aOR	Lower 95% CI	Upper 95% CI
**Sex**			
Male	1.00	reference	
Female	1.61	1.19	2.18
**Race**			
White, non-Hispanic	1.00	reference	
Black, non-Hispanic	0.89	0.64	1.23
Hispanic	1.11	0.57	2.15
Other, non-Hispanic	1.31	0.60	2.87
**Annual Household Income**			
<$15k	1.00	reference	
$15k-$24k	0.95	0.63	1.43
$25k-$34k	0.49	0.28	0.85
$35k-$49k	0.58	0.34	0.98
$50k-$74k	0.49	0.28	0.85
>$75k	0.77	0.46	1.29
**Educational Attainment**			
Did not graduate HS	1.00	reference	
Graduated HS	1.08	0.68	1.71
Some college	1.03	0.64	1.67
Graduated college	0.88	0.52	1.51
**Current smoker**			
No	1.00	reference	
Yes	1.54	1.09	2.19
**Body mass index (kg/m2)**			
<25	1.00	reference	
>=25 and <30	1.15	0.80	1.65
>=30	1.56	1.10	2.21

aOR = adjusted odds ratio; CI = confidence interval
